# *BRIP1* loss-of-function mutations confer high risk for familial ovarian cancer, but not familial breast cancer

**DOI:** 10.1186/s13058-018-0935-9

**Published:** 2018-01-24

**Authors:** Nana Weber-Lassalle, Jan Hauke, Juliane Ramser, Lisa Richters, Eva Groß, Britta Blümcke, Andrea Gehrig, Anne-Karin Kahlert, Clemens R. Müller, Karl Hackmann, Ellen Honisch, Konstantin Weber-Lassalle, Dieter Niederacher, Julika Borde, Holger Thiele, Corinna Ernst, Janine Altmüller, Guido Neidhardt, Peter Nürnberg, Kristina Klaschik, Christopher Schroeder, Konrad Platzer, Alexander E. Volk, Shan Wang-Gohrke, Walter Just, Bernd Auber, Christian Kubisch, Gunnar Schmidt, Judit Horvath, Barbara Wappenschmidt, Christoph Engel, Norbert Arnold, Bernd Dworniczak, Kerstin Rhiem, Alfons Meindl, Rita K. Schmutzler, Eric Hahnen

**Affiliations:** 1 Center for Familial Breast and Ovarian Cancer, Center for Integrated Oncology (CIO), Medical Faculty, University Hospital Cologne, Kerpener Straße 34, 50931 Cologne, Germany; 2 Department of Gynaecology and Obstetrics, Klinikum rechts der Isar der Technischen Universität München, Munich, Germany; 3 Department of Human Genetics, University Würzburg, Biozentrum, Würzburg, Germany; 4 Institute for Clinical Genetics, Technische Universität Dresden, Dresden, Germany; 5 Department of Congenital Heart Disease and Pediatric Cardiology, University Hospital Schleswig-Holstein, Campus Kiel, Kiel, Germany; 6 Department of Obstetrics and Gynecology, Medical Faculty, Heinrich-Heine-University, Düsseldorf, Germany; 7 Cologne Center for Genomics, University of Cologne, Cologne, Germany; 8 Institute of Human Genetics, University of Cologne, Cologne, Germany; 9 Cologne Excellence Cluster on Cellular Stress Responses in Aging-Associated Diseases, Cologne, Germany; 10 Institute of Medical Genetics and Applied Genomics, University Hospital Tuebingen, Tuebingen, Germany; 11 Institute of Human Genetics, University of Leipzig Hospitals and Clinics, Leipzig, Germany; 12 Institute of Human Genetics, University Medical Center Hamburg-Eppendorf, Hamburg, Germany; 13 Department of Obstetrics and Gynecology, Medical Faculty, University of Ulm, Ulm, Germany; 14 Institute of Human Genetics, University of Ulm, Ulm, Germany; 15 Institute of Human Genetics, Hannover Medical School, Hannover, Germany; 16 Department of Gynecology and Obstetrics, University Clinics Muenster, Muenster, Germany; 17 Institute for Medical Informatics, Statistics and Epidemiology (IMISE), University of Leipzig, Leipzig, Germany; 18 LIFE — Leipzig Research Centre for Civilization Diseases, University of Leipzig, Leipzig, Germany; 19 Institute of Clinical Molecular Biology, Department of Gynecology and Obstetrics, University Hospital Schleswig-Holstein, Christian-Albrechts-University of Kiel, Kiel, Germany

**Keywords:** Breast cancer, Ovarian cancer, *BRIP1* gene, Germline mutations

## Abstract

**Background:**

Germline mutations in the *BRIP1* gene have been described as conferring a moderate risk for ovarian cancer (OC), while the role of *BRIP1* in breast cancer (BC) pathogenesis remains controversial.

**Methods:**

To assess the role of deleterious *BRIP1* germline mutations in BC/OC predisposition, 6341 well-characterized index patients with BC, 706 index patients with OC, and 2189 geographically matched female controls were screened for loss-of-function (LoF) mutations and potentially damaging missense variants. All index patients met the inclusion criteria of the German Consortium for Hereditary Breast and Ovarian Cancer for germline testing and tested negative for pathogenic *BRCA1/2* variants.

**Results:**

*BRIP1* LoF mutations confer a high OC risk in familial index patients (odds ratio (OR) = 20.97, 95% confidence interval (CI) = 12.02–36.57, *P* < 0.0001) and in the subgroup of index patients with late-onset OC (OR = 29.91, 95% CI = 14.99–59.66, *P* < 0.0001). No significant association of *BRIP1* LoF mutations with familial BC was observed (OR = 1.81 95% CI = 1.00–3.30, *P* = 0.0623). In the subgroup of familial BC index patients without a family history of OC there was also no apparent association (OR = 1.42, 95% CI = 0.70–2.90, *P* = 0.3030). In 1027 familial BC index patients with a family history of OC, the *BRIP1* mutation prevalence was significantly higher than that observed in controls (OR = 3.59, 95% CI = 1.43–9.01; *P* = 0.0168). Based on the negative association between *BRIP1* LoF mutations and familial BC in the absence of an OC family history, we conclude that the elevated mutation prevalence in the latter cohort was driven by the occurrence of OC in these families. Compared with controls, predicted damaging rare missense variants were significantly more prevalent in OC (*P* = 0.0014) but not in BC (*P* = 0.0693) patients.

**Conclusions:**

To avoid ambiguous results, studies aimed at assessing the impact of candidate predisposition gene mutations on BC risk might differentiate between BC index patients with an OC family history and those without. In familial cases, we suggest that *BRIP1* is a high-risk gene for late-onset OC but not a BC predisposition gene, though minor effects cannot be excluded.

**Electronic supplementary material:**

The online version of this article (10.1186/s13058-018-0935-9) contains supplementary material, which is available to authorized users.

## Background

Monoallelic germline mutations in known predisposition genes, including *BRCA1* (OMIM 113705) and *BRCA2* (OMIM 600185), explain less than half of all cases of familial breast cancer (BC) and/or ovarian cancer (OC), and confer moderate to high risk [1–6]. In contrast to mutations in *BRCA1* and *BRCA2,* which are predisposing for both BC and OC, several risk genes appear to be tumour-site specific. Inactivating *CHEK2* (OMIM 604373) gene alterations predisposes to BC but not OC [6]. Conversely, women carrying deleterious *RAD51C* (OMIM 602774) germline mutations are at risk of developing OC [6] while an association with BC has not been established. Germline mutations in the *BRIP1* (BRCA1-interacting protein C-terminal helicase 1) gene (OMIM 605882) have been reported to confer a moderate risk for OC, especially the high-grade serous epithelial subtype [6–10], and prophylactic surgery is increasingly considered for *BRIP1* mutation carriers [11]. The impact of germline *BRIP1* mutations on BC risk, however, remains controversial.

*BRIP1* was initially described as a BC predisposition gene in 2006 [12]. The analysis of 1212 women with familial BC and 2081 control individuals revealed heterozygous truncating mutations in nine index patients and two controls, resulting in a relative risk of 2.0 (95% confidence interval (CI) = 1.2–3.2, *P* = 0.012) for BC. Buys et al. identified truncating *BRIP1* mutations in 110 of 33,767 mainly familial *BRCA1/2*-negative BC index patients, resulting in a cumulative carrier frequency of 0.33% [11], which is approximately two-fold higher than that described in the Exome Aggregation Consortium (ExAC) database (Table 1). Analysis of 1853 *BRCA1/2*-negative index patients with familial BC by Easton et al. revealed similar results, not reaching levels of significance (odds ratio (OR) = 1.62, 95% CI = 0.38–7.82, *P* = 0.45) [10]. Thompson et al. described comparable results in 2000 familial *BRCA1/2*-negative BC cases and 1997 controls (OR = 1.75, 95% CI = 0.51–5.99, *P* = 0.55) [13]. Couch et al. reported that *BRIP1* mutations confer a moderately increased risk of BC in 28,536 patients with familial and/or early-onset BC (OR = 1.63, 95% CI = 1.11–2.41, *P* = 0.01) [14]. The elevated mutation prevalence points towards *BRIP1* as a BC risk gene. This hypothesis is supported by the studies of Buys et al. and Couch et al., which showed that *BRIP1* mutation prevalence was higher among women with triple-negative breast cancer (TNBC), a tumour phenotype associated with a hereditary disease cause [11, 15, 16]. In contrast to these findings, however, *BRIP1* mutation analysis of 13,213 unselected patients with BC and 5242 control individuals by Easton et al. (SEARCH study) revealed no association with BC (OR = 0.73, 95% CI = 0.36–1.75, *P* = 0.36) [10]. Likewise, the recent analysis of Slavin et al. showed no association of truncating mutations in the *BRIP1* gene with familial BC (OR = 0.60, 95% CI = 0.10–2.33, *P* = 0.77) [17]. In summary, the role of the *BRIP1* gene in BC predisposition remains conflicting.

**Table 1:** Prevalence of heterozygous loss-of-function germline mutations in the *BRIP1* gene in control cohorts and BC/OC index patients according to tumour site, family history, and age at first diagnosis

Study sample	*n*	Negative	Positive (%)	OR	95% CI	*P* value^a^	Mean AAD (range)
ExAC control database	27,173	27,135	38 (0.14)	–	–	–	–
FLOSSIES control database	7325	7316	9 (0.12)	–	–	–	–
Geographically matched controls	2189	2186	3 (0.14)	–	–	–	–
All controls	36,687	36,637	50 (0.14)	–	–	–	–
OC index patients	706	688	18 (2.55)	19.17	11.13–33.03	<0.0001	54 (20–93)^b^
Affected by OC only	523	507	16 (3.06)	23.12	13.08–40.88	< 0.0001	53 (20–93)^c^
Affected by OC and BC	183	181	2 (1.09)	8.10	1.96–33.53	0.0276	60 (26–83)^d^
AAD OC < 51 years	246	244	2 (0.81)	6.01	1.45–24.82	0.0471	39 (20–50)
AAD OC < 61 years	425	417	8 (1.88)	14.06	6.62–29.84	< 0.0001	46 (20–60)
AAD OC ≥ 61 years	255	245	10 (3.92)	29.91	14.99–59.66	< 0.0001	69 (60–93)
Familial OC index cases, overall	611	594	17 (2.78)	20.97	12.02–36.57	< 0.0001	54 (20–93)^e^
Familial OC index cases, relative(s) with BC only	421	412	9 (2.14)	16.01	7.82–23.76	< 0.0001	53 (20–85)^f^
Familial OC index cases, relative(s) with OC	190	182	8 (4.21)	32.21	15.06–68.90	< 0.0001	54 (21–93)^g^
BC index patients	6341	6325	16 (0.25)	1.85	1.06–3.26	0.0363	47 (17–92)
AAD BC < 51 years	4417	4407	10 (0.23)	1.66	0.84–3.28	0.1424	41 (17–50)
AAD BC < 61 years	5627	5612	15 (0.27)	1.96	1.10–3.49	0.0272	44 (17–60)
AAD BC ≥ 61 years	714	713	1 (0.14)	1.03	0.14–7.45	0.6260	68 (61–92)
Familial BC index cases, overall	5668	5654	14 (0.25)	1.81	1.00–3.30	0.0623	48 (17–92)
Familial BC index patients, relative(s) with BC only^h^	4641	4632	9 (0.19)	1.42	0.70–2.90	0.3030	48 (17–92)
Familial BC index patients, relative(s) with OC^i^	1027	1022	5 (0.49)	3.59	1.43–9.01	0.0168	50 (17–92)

Univariate logistic regression was performed to estimate odds ratios (OR) and 95% confidence interval (CI)

*AAD* age at first diagnosis, *BC* breast cancer, *ExAC* Exome Aggregation Consortium, *OC* ovarian cancer

^a^Fisher’s exact test

^b–g^Mean AAD and range refers to a subgroup of 680 patients (^b^), 511 patients (^c^), 169 patients (^d^), 593 patients (^e^), 410 patients (^f^), and 183 patients (^g^)

^h^2238 BC index patients had one relative with BC and 2403 BC index patients had at least two relatives with BC

^i^ All BC index patients reported at least one relative with OC. In addition, 382 BC index patients had no relatives with BC, 282 BC index cases described one relative with BC, and 363 BC index patients two relatives with BC

This case-control study aimed to further elucidate the role of *BRIP1* in cancer predisposition by analysing its coding region (transcript NM_032043.2) in a well-characterized sample of 6341 BC and 706 OC index patients of German descent, along with 2189 geographically matched female control individuals. We suggest that the elevated *BRIP1* mutation prevalence described in some studies with the focus on familial BC might be due to the co-occurrence of OC in these families, one generally used criterion to define a positive cancer family history. To circumvent sample selection bias, we stratified our BC study sample by the familial occurrence of OC.

## Methods

### Study cohorts

All index patients were female and met the inclusion criteria of the German Consortium for Hereditary Breast and Ovarian Cancer (GC-HBOC) for germline testing (Additional file 1: Table S1). The GC-HBOC inclusion criteria are not restricted to familial cases and also consider patients with early-onset BC (age at first diagnosis (AAD) before 36 years), bilateral BC (AAD before 51 years), and patients affected by BC and OC even in the absence of a family history of BC and OC (Additional file 1: Table S1). Index patients with at least one relative affected by BC or OC were defined as familial index patients. Written informed consent was obtained from all patients, and ethical approval was granted by the Ethics Committee of the University of Cologne (07-048). All patients tested negative for pathogenic *BRCA1/2* mutations. In addition, we analysed 2189 geographically matched female control individuals (GMCs). All GMCs were cancer-free and aged 40 years or above at the time of the blood draw (mean age ± SD, 63 ± 10 years). The cancer family history of the GMCs is undocumented. In this case-control investigation we additionally employed two publicly accessible control datasets (ExAC and FLOSSIES). The FLOSSIES database includes gene panel sequence data of 7325 women of European American ancestry who are cancer-free until at least 70 years of age (cancer family history undocumented). The ExAC dataset comprises whole exome sequencing data of 27,173 individuals of non-Finnish European ancestry (excluding The Cancer Genome Atlas data). For ExAC, neither personal nor family cancer histories are publicly available.

### Next-generation sequencing

All index patients and GMCs were screened for germline mutations in the *BRIP1* gene (transcript NM_032043.2) by next-generation sequencing (NGS) using blood-derived DNA samples. NGS was performed at each participating centre using Illumina sequencing devices (MiSeq, NextSeq) employing either the customized TruRisk® (GC-HBOC designed; manufactured by Agilent or Illumina) or the TruSight™ Cancer gene panel (Illumina) for hybrid capture target enrichment. Bioinformatic processing of the data was performed using JSI Medical Systems Sequence Pilot, Sophia Genetics DDM, or similar software packages certified for clinical diagnostics. The diagnostic pipelines of the laboratories involved have been successfully tested in European Molecular Genetics Quality Network (EMQN) schemes.

### Variant classification

Variant classification was performed in accordance with the regulations of the international ENIGMA consortium (Evidence-based Network for the Interpretation of Germline Mutant Alleles; https://enigmaconsortium.org; version 1.1: 26 March 2015). All genetic variants were classified using a five-tier variant classification system as proposed by the International Agency for Research on Cancer (IARC) Unclassified Genetic Variants Working Group. All class 4/5 *BRIP1* mutations identified by NGS were verified by standard Sanger sequencing. Truncating variants were defined as stop-gain, frameshift, or essential splice-site mutations affecting invariant splice sites or the last nucleotide of an exon. Loss-of-function (LoF) variants were defined as truncating variants not affecting the last exon of the *BRIP1* gene (exon 20). For the identification of potentially damaging, rare missense variants we employed two in silico prediction tools (SIFT and MutationTaster). Missense variants were defined as potentially damaging when predicted deleterious by the in silico tools SIFT and MutationTaster (Alamut version 2.10 as 9 November 2017).

## Results

All truncating mutations identified in the *BRIP1* gene are listed in (Additional file 1: Table S2). The analysis of 706 OC index patients revealed 18 LoF mutation carriers, resulting in a cumulative carrier frequency of 2.55%. Based on whole-exome sequencing data provided by the ExAC [18], 0.14% of the individuals of non-Finnish European origin carried heterozygous LoF mutations within the *BRIP1* gene (excluding The Cancer Genome Atlas data). This frequency is similar to that observed in the FLOSSIES database (https://whi.color.com). Of the 7325 women with American-European ancestry who remained cancer-free until at least 70 years of age, 9 (0.12%) carried heterozygous *BRIP1* LoF mutations. Among the 2189 GMCs screened in our study, 3 LoF mutation carriers were identified (cumulative carrier frequency, 0.14%; Table 1). The comparison of the *BRIP1* mutation prevalence in OC index patients and all controls revealed an OR of 19.17 (95% CI = 11.13–33.03, *P* < 0.0001; Table 1).

In the overall cohort of 706 OC index patients, 523 patients were affected by OC only and 183 by OC and BC. When stratified for personal cancer history, an OR of 23.12 (95% CI = 13.08–40.88, *P* < 0.0001; Table 1) was observed in the subgroup of patients affected by OC only, which is considerably higher than the association observed in patients with OC and BC (OR = 8.10, 95% CI = 1.96.08–33.53, *P* = 0.0276; Table 1). The mean AAD of OC was reported for 680 out of 706 patients, with a mean of 54 years (range 20–93 years; Table 1). When stratified for AAD, the *BRIP1* mutation prevalence rose with increasing AAD. While the association of *BRIP1* LoF mutations with early-onset OC (AAD < 51 years) was barely significant (OR = 6.01, 95% CI = 1.45–24.82, *P* = 0.0471), a high OR of 29.91 (95% CI = 14.99–59.66, *P* < 0.0001) was observed in OC patients with an AAD ≥ 61 years. Of the 706 OC index patients, 611 OC index patients show a BC and/or OC cancer family history. In familial OC index cases, an OR of 20.97 was observed (95% CI = 12.02–36.57, *P* < 0.0001; Table 1). Of note, familial OC index patients with an additional OC family history show a considerably higher *BRIP1* mutation prevalence (OR = 32.21, 95% CI = 15.06–68.90, *P* < 0.0001; Table 1) than familial OC index patients with a BC-only family history (OR = 16.01, 95% CI = 7.82–23.76, *P* < 0.0001; Table 1).

In summary, *BRIP1* appears to be a high-risk gene for late-onset familial OC. All *BRIP1* mutation carriers tested negative for further pathogenic mutations in BC/OC predisposition genes (Additional file 1: Table S3). Most OC patients with *BRIP1* mutations (mean AAD OC 61 years, range 26–76 years) show a high-grade serous tumour phenotype (Additional file 1: Table S3), consistent with published results [6, 8]. Importantly, the OC patient with an AAD of 26 years developed endometrioid OC, a comparatively rare OC subtype [19]. Following standard therapy, the patient remained cancer-free (last medical examination at 46 years). Large genomic rearrangements affecting the *EPCAM* gene were additionally excluded in this patient (data not shown).

In the overall sample of 6341 BC index patients, 16 mutation carriers were observed, resulting in a cumulative carrier frequency of 0.25% (OR = 1.85, 95% CI = 1.06–3.26, *P* = 0.0363). Most mutation carriers with BC developed hormone receptor-positive BC (Additional file 1: Table S3). When stratified for AAD, a barely significant association of *BRIP1* LoF mutations with BC was observed in the subgroup of patients with an AAD < 61 years (Table 1). Of the 6341 BC index patients, 5668 BC index patients show a BC and/or OC cancer family history. No significant association between *BRIP1* LoF mutations and familial BC was observed (OR = 1.81, 95% CI = 1.00–3.30, *P* = 0.0623; Table 1). In the BC sample comprising 4641 familial BC index patients without a personal or familial OC history, nine patients carried heterozygous *BRIP1* LoF mutations, resulting in a cumulative carrier frequency of 0.19% (OR = 1.42, 95% CI = 0.70–2.90, *P* = 0.3030; Table 1). Analysis of 1027 familial BC index patients with a family history of OC, however, revealed different results. In this cohort, we identified five patients carrying LoF mutations (cumulative carrier frequency, 0.49%; Table 1). This frequency is significantly higher than that observed in controls (OR = 3.59, 95% CI = 1.43–9.01, *P* = 0.0168; Table 1). Based on the overall negative association between *BRIP1* LoF mutations and familial BC, we conclude that this elevated mutation prevalence was mainly driven by the familial occurrence of OC, rather than by a predisposing role of *BRIP1* mutations in BC pathogenesis.

Data on proven deleterious *BRIP1* missense mutations are sparse. However, Ramus et al. demonstrated an association with OC for rare *BRIP1* missense variants (minor allele frequency (MAF) < 1%) that were predicted damaging by in silico tools such as SIFT and MutationTaster [8]. In controls (ExAC, FLOSSIES, GMCs), the cumulative carrier frequency for rare *BRIP1* missense variants (MAF < 1%) predicted damaging by both SIFT and MutationTaster was 1.32% (485 of 36,687; Additional file 1: Table S4). Compared with controls, rare *BRIP1* missense variants predicted damaging by both tools were significantly more prevalent in OC patients (2.83%, 20 of 706; *P* = 0.00139), but not in BC patients (Additional file 1: Table S4).

## Discussion

To avoid ambiguous results, studies aimed at assessing the impact of candidate predisposing gene mutations on BC risk might differentiate between BC index patients with a family history of OC and those without. In this study, LoF mutations in the *BRIP1* gene were not statistically associated with familial BC (Table 1). Although we analysed a large series of index patients with BC, minor effects of *BRIP1* mutations on BC risk cannot be fully excluded, and this requires further investigation.

In study samples selected for cancer family history, for example, the mutation prevalence of a risk gene is generally higher than in unselected cases. The AGO-TR-1 trial, for example, revealed pathogenic *BRCA1/2* variants in 109 out of 523 unselected patients with OC (20.8%), while 71 *BRCA1/2* mutation carriers were observed in the subgroup of 225 familial cases (31.6%) [19]. In our study, mainly focussing on index patients with familial OC, the *BRIP1* mutation prevalence (cumulative carrier frequency, 2.78%; Table 1) is considerably higher than in studies focussing on unselected patients with OC. Norquist et al. [6] identified *BRIP1* LoF mutations in 23 out of 1915 unselected patients with OC (cumulative carrier frequency, 1.20%). Kurian and colleagues [20] analysed 5020 patients with OC, most of whom were not showing a positive family history. In this unselected sample of OC patients, 36 *BRIP1* mutation carriers were observed (cumulative carrier frequency, 0.72%). Ramus et al. [8] identified 30 truncating *BRIP1* mutations in 3257 patients (cumulative carrier frequency, 0.92%) and three truncating mutations in 3444 controls (cumulative carrier frequency, 0.09%).

Age-dependent disease risks cannot be calculated solely based on case-control data generated in our study. For *BRIP1* mutation carriers, Ramus et al. calculated a cumulative OC risk by age 80 of 5.8% (95% CI = 3.6–9.1%), possibly warranting risk-reducing salpingo-oophorectomy (RRSO) [8]. The highly significant associations shown in our study suggest that *BRIP1* represents a high-risk gene for late-onset OC, further supporting the notion that RRSO should be considered for *BRIP1* mutation carriers. For *BRIP1* mutation carriers, we observed a mean AAD of 61 years (range 26–76 years), which tended to be older than in the overall sample of familial OC index patients (mean AAD 54 years, range 20–93 years; Table 1). The mean AAD for *BRIP1* mutation carriers described in our study was comparable with the data presented by Norquist et al. [6] (mean AAD 62 years, range 43–79 years) and Ramus et al. [8] (mean AAD 64, range 47–82 years).

This study has several limitations. The cohort of familial OC index patients is comparatively small, and results should be validated in larger studies focussing on familial OC patients. Moreover, we focussed on truncating variants not affecting the last exon of the *BRIP1* gene. Truncating last exon variants in the *BRIP1* gene, which may or may not impair protein function, were present in 0.06% of the ExAC controls (Additional file 1: Table S2), in one familial OC index patient (0.16%), and in two familial BC index patients without OC family history (0.04%). Thus, the inclusion of truncating last exon variants would marginally change the calculated ORs, but not our main conclusions.

## Conclusions

*BRIP1* LoF mutations confer a high OC risk in familial index patients (OR = 20.97, 95% CI = 12.02–36.57, *P* < 0.0001) and in the subgroup of index patients with late-onset OC (OR = 29.91, 95% CI = 14.99–59.66, *P* < 0.0001). No significant association between *BRIP1* LoF mutations and familial BC was observed (OR = 1.81, 95% CI = 1.00–3.30, *P* = 0.0623), although minor effects cannot be excluded. For OC, the highly significant associations shown in our study suggest that *BRIP1* represents a high risk rather than a moderately penetrant predisposition gene, further supporting the notion that RRSO should be considered for *BRIP1* mutation carriers.

## Additional file

Additional file 1: Table S1. Inclusion criteria of the German Consortium for Hereditary Breast and Ovarian Cancer (GC-HBOC) for *BRCA1* and *BRCA2* germline testing. **Table S2.** Heterozygous protein-truncating mutations identified in the *BRIP1* gene. **Figure S1.** Characterization of the c.507G > A variant within the *BRIP1* gene (rs876660937) on transcript level. **Table S3.** Genotypes and phenotypes of heterozygous *BRIP1* mutation carriers identified within the BC/OC index patient cohorts. **Table S4.** Potentially damaging missense variants identified in the *BRIP1* gene. (PDF 215 kb)

## Abbreviations

AAD: Age at first diagnosis
BC: Breast cancer
CI: Confidence interval
ExAC: Exome Aggregation Consortium
GC-HBOC: German Consortium for Hereditary Breast and Ovarian Cancer
GMC: Geographically matched control individual
LoF: Loss-of-function
MAF: Minor allele frequency
NGS: Next-generation sequencing
OC: Ovarian cancer
OR: Odds ratio
RRSS: Risk-reducing salpingo-oophorectomy
SD: Standard deviation
TNBC: Triple-negative breast cancer
