# Supplementary material for: BRIP1 loss-of-function mutations confer high risk for familial ovarian cancer, but not familial breast cancer
Source: Breast Cancer Res. 2018 Jan 24;20:7. doi: 10.1186/s13058-018-0935-9 (PMC5784717; doi:10.1186/s13058-018-0935-9)
Supplement: Additional file 1: Table S1. — Inclusion criteria of the German Consortium for Hereditary Breast and Ovarian Cancer (GC-HBOC) for BRCA1 and BRCA2 germline testing. Table S2. Heterozygous protein-truncating mutations identified in the BRIP1 gene. Figure S1. Characterization of the c.507G > A variant within the BRIP1 gene (rs876660937) on transcript level. Table S3. Genotypes and phenotypes of heterozygous BRIP1 mutation carriers identified within the BC/OC index patient cohorts. Table S4. Potentially damaging missense variants identified in the BRIP1 gene. (PDF 215 kb) [file 13058_2018_935_MOESM1_ESM.pdf]

BRIP1 loss-of-function mutations confer high risk for familial ovarian cancer, but not familial breast cancer

Online Only Supplemental Material:

Table S1: Inclusion criteria for germline testing

Table S2: Truncating germline mutations identified in the *BRIP1* gene

Figure S1: Characterization of the c.507G>A variant in the *BRIP1* gene

Table S3: Genotypes and phenotypes of heterozygous *BRIP1* germline mutation carriers

Table S4: Potentially damaging missense variants identified in the *BRIP1* gene

Table S1: Inclusion criteria of the German Consortium for Hereditary Breast and Ovarian Cancer (GC-HBOC) for *BRCA1* and *BRCA2* germline testing. Ductal carcinoma in situ (DCIS) is categorized as breast cancer.

- ≥3 women with breast cancer
- ≥2 women with breast cancer, 1 with onset below 51 years of age
- ≥1 woman with breast cancer and 1 woman with ovarian cancer
- ≥2 women with ovarian cancer
- ≥1 woman with breast- and ovarian cancer
- ≥1 woman with breast cancer below 36 years of age
- ≥1 woman with bilateral breast cancer with onset below 51 years
- ≥1 male with breast cancer and 1 woman with breast- or ovarian cancer

genomic position (GRChr37/hg19)	cDNA position (NM_032043.2)	protein	exon	rs-number	carrier frequency ExAC (total)	carrier frequency ExAC (%)	carrier frequency FLOSSIES (total)	carrier frequency FLOSSIES (%)	carrier frequency GMCs (total)	carrier frequency GMCs (%)	carrier frequency index patients (total)	carrier frequency index patients (%)
truncating mutations (exons 1-19)												
17:59763337A>C	c.2765T>G	p.(Leu922Ter)	19/20	rs587782410	1/27,170	0.004	/	/	/	/	1/7,047	0.014
17:59763370dup	c.2732dup	p.(Thr912AspfsTer27)	19/20	rs752780954	6/27,172	0.022	/	/	/	/	/	/
17:59763415_59763418del	c.2684_2687del	p.(Ser895Ter)	19/20	rs760551339	1/27,171	0.004	/	/	/	/	/	/
17:59763496del	c.2606del	p.(Gln869ArgfsTer28)	19/20	/	/	/	/	/	1/2,189	0.047	/	/
17:59793404G>C	c.2400C>G	p.(Tyr800Ter)	17/20	rs574552037	2/27,013	0.007	1/7,325	0.014	1/2,189	0.047	8/7,047	0.114
17:59793412G>A	c.2392C>T	p.(Arg798Ter)	17/20	rs137852986	9/26,844	0.034	5/7,325	0.068	/	/	3/7,047	0.043
17:59820480dup	c.2273dup	p.(Ala759SerfsTer6)	16/20	rs587780236	/	/	/	/	/	/	2/7,047	0.028
17:59821794_59821795del	c.2255_2256del	p.(Lys752ArgfsTer12)	15/20	rs730881649	/	/	/	/	/	/	1/7,047	0.014
17:59821941_59821942insGG	c.2108_2109insCC	p.(Lys703AsnfsTer3)	15/20	rs760863397	2/27,142	0.007	1/7,325	0.014	/	/	/	/
17:59853806G>A	c.2053C>T	p.(Gln685Ter)	14/20	rs876659533	/	/	/	/	/	/	1/7,047	0.014
17:59853849dup	c.2010dup	p.(Glu671Ter)	14/20	rs775537066	2/27,172	0.007	/	/	/	/	1/7,047	0.014
17:59853889del	c.1970del	p.(Gly657ValfsTer31)	14/20	rs760782298	2/27,170	0.007	/	/	/	/	/	/
17:59857669dup	c.1888dup	p.(Thr630AsnfsTer9)	13/20	rs763818712	1/27,160	0.004	/	/	/	/	/	/
17:59857753dup	c.1803_1804insG	p.(Asp602GlyfsTer3)	13/20	/	/	/	/	/	/	/	1/7,047	0.014
17:59857764T>A	c.1795-2A>T	p.(?)	13/20	rs775066436	2/27,104	0.007	/	/	/	/	/	/
17:59858204del	c.1791del	p.(Val598TrpfsTer15)	12/20	/	/	/	/	/	/	/	1/7,047	0.014
17:59858254G>A	c.1741C>T	p.(Arg581Ter)	12/20	rs780020495	/	/	/	/	/	/	2/7,047	0.028
17:59861749dup	c.1510dup	p.(Ile504AsnfsTer7)	11/20	rs775735278	/	/	/	/	/	/	1/7,047	0.014
17:59861784_59861785del	c.1474_1475del	p.(Gly492ThrfsTer18)	11/20	/	/	/	1/7,325	0.014	/	/	/	/
17:59870957C>A	c.1473+1G>T	p.?	10/20	rs748274524	1/15,371	0.007	/	/	/	/	/	/
17:59871048A>C	c.1383T>G	p.(Tyr461Ter)	10/20	rs587780875	/	/	/	/	/	/	1/7,047	0.014
17:59871059C>A	c.1372G>T	p.(Glu458Ter)	10/20	rs587780228	1/22,443	0.004	/	/	/	/	/	/
17:59876561G>A	c.1240C>T	p.(Gln414Ter)	09/20	rs368796923	1/27,146	0.004	/	/	/	/	/	/
17:59876565del	c.1236del	p.(Val413PhefsTer10)	09/20	rs863224525	/	/	/	/	/	/	1/7,047	0.014
17:59878653A>T	c.1101T>A	p.(Cys367Ter)	08/20	/	/	/	/	/	/	/	1/7,047	0.014
17:59885827C>T	c.918+1G>A	p.(?)	07/20	rs587781655	/	/	/	/	/	/	1/7,047	0.014
17:59886113del	c.633del	p.(Gly212AlafsTer62)	07/20	rs779466229	3/27,005	0.011	/	/	/	/	/	/
17:59924461C>T	c.627+1G>A	p.(?)	06/20	rs587780833	/	/	/	/	/	/	1/7,047	0.014
17:59926490C>T	c.507G>A[#]	r.380_507del; p.(Ser128Ter)	05/20	rs876660937	/	/	/	/	/	/	5/7,047	0.071
17:59926606T>A	c.391A>T	p.(Lys131Ter)	05/20	/	/	/	/	/	/	/	1/7,047	0.014
17:59934594A>G	c.206-2A>G	p.(?)	04/20	rs786203700	/	/	/	/	1/2,189	0.047	/	/
17:59937169C>T	c.193C>T	p.(Gln65Ter)	03/20	rs575595017	3/27,149	0.011	/	/	/	/	/	/
17:59937174G>A	c.188G>A	p.(Trp63Ter)	03/20	/	/	/	1/7,325	0.014	/	/	/	/
17:59938846dup	c.55dup	p.(Tyr19LeufsTer2)	02/20	/	/	/	/	/	/	/	1/7,047	0.014
17:59938877T>A	c.24T>A	p.(Tyr8Ter)	02/20	rs752411477	1/27,118	0.004	/	/	/	/	/	/
**Cumulative Carrier Frequency (%)** truncating mutations						**0.140**		**0.123**		**0.137**		**0.482**

(exon 20)												
17:59760756C>T	c.3651G>A	p.(Trp1217Ter)	20/20	rs542698396	3/27075	0.011	/	/	/	/	/	/
17:59760882dup	c.3525dup	p.(Ile1176TyrfsTer13)	20/20	rs777367075	1/27,060	0.004	/	/	/	/	/	/
17:59760967dup	c.3440dup	p.(Asn1147LysfsTer2)	20/20	rs753683450	2/27,079	0.007	/	/	/	/	/	/
17:59761006del	c.3401del	p.(Pro1134LeufsTer16)	20/20	rs756853672	1/27,108	0.004	/	/	/	/	/	/
17:59761014_59761017del	c.3390_3393del	p.(Tyr1131LeufsTer18)	20/20	rs778664039	5/27,107	0.018	/	/	/	/	/	/
17:59761147dup	c.3260dup	p.(Asn1087LysfsTer4)	20/20	rs771654971	1/27,173	0.004	/	/	/	/	/	/
17:59761321del	c.3086del	p.(Ser1029MetfsTer30)	20/20	rs773433456	1/27,173	0.004	/	/	/	/	/	/
17:59761412_59761415del	c.2992_2995del	p.(Lys998GlufsTer60)	20/20	rs786203717	/	/	/	/	/	/	2/7,047	0.028
17:59761414_59761417dup	c.2990_2993dup	p.(Lys998AsnfsTer5)	20/20	rs878855150	/	/	2/7,325	0.027	/	/	1/7,047	0.014
17:59761414_59761417del	c.2990_2993del	p.(Thr997ArgfsTer61)	20/20	rs771028677	2/27,149	0.007	/	/	/	/	/	/
**Cumulative Carrier Frequency (%)**						**0.059**		**0.027**		**0.000**		**0.043**

Table S2: Heterozygous protein-truncating mutations identified in the *BRIP1* gene (transcript NM_032043.2). Truncating mutations were defined as nonsense, frameshift and essential splice-site mutations (affecting invariant splice sites or the last nucleotide of an exon). Of note, only truncating mutations affecting exons 1-19 of the *BRIP1* gene were defined as loss of function (LoF) mutations. Variants in the last exon (20) of the *BRIP1* gene were not considered LoF because of their questionable relevance concerning pathogenicity. A total of 16 distinct germline LoF mutations were listed in the ExAC database (Exome Aggregation Consortium, non-Finnish Europeans; excluding The Cancer Genome Atlas data; as of June 2016); 5 were listed in the FLOSSIES database (American-European ancestry) and 3 were identified in geographically-matched female controls (GMCs); 19 distinct LoF mutations were found in 7,047 BC/OC index patients. All LoF mutations identified in index patients and GMCs were verified by standard Sanger sequencing. Our analysis did not cover copy number variations (CNVs). CNVs reported in the ExAC/FLOSSIES databases were excluded. / = variant not present in cohort. # = The variant c.507G>A affects the last nucleotide of exon 5 and was predicted spliceogenic by MaxEntScan (data not shown). We experimentally demonstrate that this variant causes a complete skipping of exon 5, resulting in a frameshift and premature stop codon (Figure S1 in the Supplement).

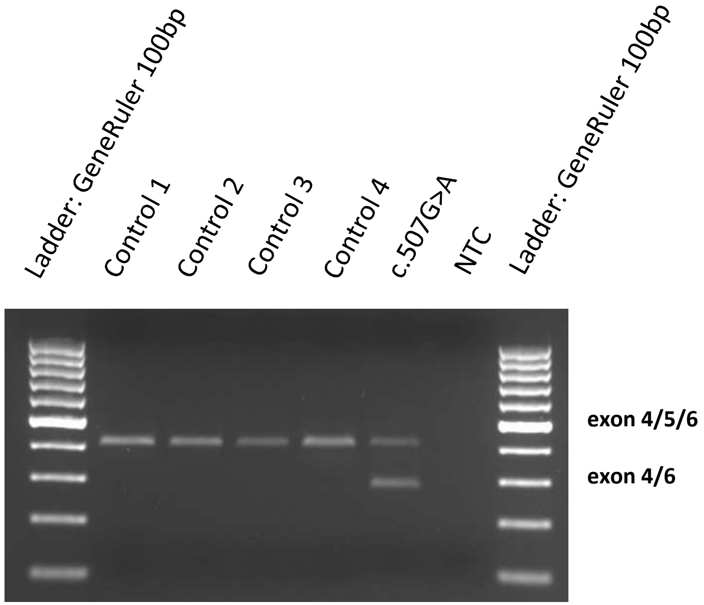

Figure S1: Characterization of the c.507G>A variant within the *BRIP1* gene (rs876660937) on transcript level. Total RNA was isolated using the PaxGene Blood RNA Kit 50 v.2 from PreAnalytiX (Qiagen BD Company, Lot-Nr: 157014022). 500ng of total RNA was reverse transcribed by employing the Transcriptor High Fidelity cDNA synthesis Kit (Roche Applied Science, Mannheim, Germany) and anchored-oligo $(dT)_{18}$ primers. RNA concentrations were measured using a NanoDrop ND 1000 spectrophotometer. The following oligonucleotides were used for target amplification: exon_4_forward: GCAGATGAGGGCGTAAGTG and exon_6_reverse TTCTGTGGCGAAAAGGAGTT. PCR products were separated by a 2% agarose gel electrophoresis. The c.507G>A variant caused skipping of exon 5, resulting in a product which is 127 nucleotides shorter than the full-length product (412 nucleotides). The wild-type (WT)-sized cDNA band was extracted and subjected to Sanger sequencing. No heterozygous signal was observed at position c.507 (data not shown), suggesting complete skipping of exon 5.

patient	*BRIP1* mutation	cancer site (age at first diagnosis, index patient)	family history	tumour histology	grading	ER Status	PR status	HER2 status	TNBC
**OC index patients**									
1	c.1803_1804insG, p.(Asp602fsTer3)	OC(26)	1xOC(26), 3xBC(42,65,63)	endometrioid	G2				
2	c.1101T>A, p.(Cys367Ter)	OC(48)	2xBC(43,82), 1xOC(48)	high grade serous	G3				
3	c.2392C>T, p.(Arg798Ter)	OC(57)	1xBC (>51), 1xOC(57)	high grade serous	G3				
4	c.1383T>G, p.(Tyr461Ter)	OC(58)	2xOC(58,/), 2xBC(50,75)	high grade serous	G3				
5	c.507G>A, , r.380_507del; p.(Ser128Ter)	OC(59)	2xOC(44,59)	/	/				
6	c.2400C>G, p.(Tyr800Ter)	OC(59)	1xOC(59), BC(50)	undifferentiated	G4				
7	c.2255_2256del, p.(Lys752ArgfsTer12)	OC(59)[#]	1xOC(59)[##]	high grade serous	G3				
8	c.507G>A, r.380_507del; p.(Ser128Ter)	OC(60)	1xBC(35), 2xOC(60,65)	high grade serous	G3				
9	c.507G>A, r.380_507del; p.(Ser128Ter)	OC(62)	1xBC(/), 1xOC(62)	high grade serous	G3				
10	c.627+1G>A, p.(?)	OC(62)[##]	1xOC(62)[#], 1xBC(51)	high grade serous	G3				
11	c.2400C>G, p.(Tyr800Ter)	OC(63)	1xOC(63), 1xBC(<50)	high grade serous	G3				
12	c.2273dup, p.(Ala759SerfsTer6)	OC(63)	2xOC(63,58)	/	/				
13	c.2010dup, p.(Glu671Ter)	OC(64)	1xOC(64), 1xBC/OC(54,60), 2xBC(80,55)	high grade serous	G3				
14	c.55dup, p.(Tyr19LeufsTer2)	OC(66)	1xBC(63), 2xOC(66,68)	low grade serous	G2				
15	c.2053C>T, p.(Gln685Ter)	OC(72)	1xBC(42), 1xOC(72)	high grade serous	G3				
16	c.507G>A, p.(Gln169Gln)	OC(73)	2xOC(73,73), 1xBC(70)	low grade serous	G1				
17	c.1236del, p.(Val413PhefsTer10)	OC(75)	2xOC (75,71)	low grade serous	G1				
18	c.2392C>T, p.(Arg798Ter)	OC(76)	2xBC(40,60), 1xOC(76)	high grade serous	G3				
**BC index patients**									
1	c.1510dup, p.(Ile504AsnfsTer7)	BC(30)	1xBC(30)	non-special type	G3	pos	pos	pos	no
2	c.2400C>G, p.(Tyr800Ter)	BC(32)	2xBC(32,92)	non-special type	G3	pos	pos	neg	no
3	c.2400C>G, p.(Tyr800Ter)	BC(35)	1xBC(35)	non-special type+DCIS	G2	pos	pos	neg	no
4	c.2765T>G, p.(Leu922Ter)	BC(42)	2xBC(42,48)	non-special type	G2	pos	pos	neg	no
5	c.507G>A, p.(Gln169Gln)	BCbil(44,65)	3xBC(44,65,>50)	/<br>non-special type	/<br>G3	/<br>neg	/<br>neg	/<br>neg	/<br>yes
6	c.2400C>G, p.(Tyr800Ter)	BC(45)	2xBC(45,50), 1xOC(50)	/	/	/	/	/	/
7	c.1741C>T, p.(Arg581Ter)	BC(46)	2xBC(46,75)	DCIS	/	pos	pos	neg	no
8	c.2273dup, p.(Ala759SerfsTer6)	BC(48)	1xBC(48), 1xOC(67)	non-special type	G3	pos	pos	neg	no
9	c.2400C>G, p.(Tyr800Ter)	BC(49)	4xBC(46,49,50,57)	DCIS	/	/	/	/	/
10	c.1741C>T, p.(Arg581Ter)	BC(49)	2xBC(49,70)	medullary	G3	neg	neg	pos	no
11	c.2392C>T, p.(Arg798Ter)	BC(54)	2xBC(54,>50)	non-special type	G3	neg	neg	neg	yes
12	c.391A>T, p.(Lys131Ter)	BC(56)	1xBC(56), 1xOC(81)	lobular	G2	neg	neg	pos	no
13	c.1791del, p.(Val598TrpfsTer15)	BC(56)	3xBC(43,52,56)	non-special type	G2	pos	pos	neg	no
14	c.918+1G>A, p.(?)	BC(59)	1xBC(51), 1xOC(/)	non-special type	G3	neg	neg	neg	yes
15	c.2400C>G, p.(Tyr800Ter)	BCbil(60,66)	3xBC(60,66,40)	non-special type+DCIS<br>non-special type+DCIS	G2<br>G2	neg<br>pos	neg<br>pos	pos<br>neg	no<br>no
16	c.2400C>G, p.(Tyr800Ter)	BC(62)	2xBC(50,62), 1xOC(55)	invasive lobular	G2	pos	pos	neg	no

Table S3: Genotypes and phenotypes of heterozygous *BRIP1* mutation carriers identified within the BC/OC index patient cohorts. All *BRIP1* mutation carriers were negative for class 4/5 variants in 14 genes analysed (*ATM, BRCA1, BRCA2, CHEK2, PALB2, RAD51C, RAD51D, TP53, CDH1, NBN, MSH2, MSH6, MLH1, PMS2*). Given are the cancer sites and age at first diagnosis, family history (including index patient), tumour histology, grading, and receptor status for BC. ER = estrogen receptor, PR = progesterone receptor, HER2 = human epidermal growth factor receptor 2, TNBC = triple negative breast cancer, / = data not available. [#] = Patient developed bilateral BC at the age of 38 (G2, ER positive, PR positive, HER2 not determined) and 42 (DCIS). [# #]= Patient developed BC at the age of 66 years (G1, ER positive, PR negative, HER2 negative).

genomic position (GRChr37/hg19)	cDNA position (NM_032043.2)	protein	exon position	rs-number	carrier frequency ExAC (n=27,173)	carrier frequency FLOSSIES (n=7,325)	carrier frequency GMCs (n=2,189)	carrier frequency all controls (n=36,687)	carrier frequency OC index patients (n=706)	carrier frequency BC index patients (n=6,341)
17:59760679C>A	c.3728G>T	p.(Gly1243Val)	20/20	rs765545033	1	0	0	1	0	0
17:59760997T>C	c.3410A>G	p.(Tyr1137Cys)	20/20	/	0	0	0	0	0	1
17:59761004C>T	c.3403G>A	p.(Glu1135Lys)	20/20	rs369340444	1	0	0	1	0	0
17:59761009G>A	c.3398C>T	p.(Thr1133Ile)	20/20	/	0	0	1	1	0	0
17:59761444G>A	c.2963C>T	p.(Ser988Phe)	20/20	rs758032378	1	0	0	1	0	0
17:59763509G>A	c.2593C>T	p.(Arg865Trp)	19/20	rs578022079	0	2	0	2	0	1
17:59763520G>C	c.2582C>G	p.(Ser861Cys)	19/20	rs774415723	1	0	0	1	0	0
17:59770823C>T	c.2543G>A	p.(Arg848His)	18/20	rs374334794	0	0	0	0	1	0
17:59770868A>G	c.2498T>C	p.(Ile833Thr)	18/20	rs876660936	0	0	0	0	4	1
17:59793327T>C	c.2477A>G	p.(Asn826Ser)	17/20	rs760127237	1	0	0	1	0	0
17:59793335C>A	c.2469G>T	p.(Arg823Ser)	17/20	rs587780239	3	1	0	4	0	0
17:59793340A>G	c.2464T>C	p.(Tyr822His)	17/20	rs760887592	0	0	0	0	0	1
17:59793381C>A	c.2423G>T	p.(Arg808Ile)	17/20	rs781153382	1	0	0	1	0	0
17:59793398G>T	c.2406C>A	p.(Asp802Glu)	17/20	rs748981650	1	0	0	1	0	0
17:59793414T>C	c.2390A>G	p.(Lys797Arg)	17/20	rs730881622	0	1	0	1	0	0
17:59820423C>T	c.2330G>A	p.(Arg777His)	16/20	rs747568830	1	0	0	1	0	0
17:59820424G>C	c.2329C>G	p.(Arg777Gly)	16/20	rs768555161	3	0	0	3	0	0
17:59820436C>T	c.2317G>A	p.(Asp773Asn)	16/20	rs146091205	1	0	0	1	0	0
17:59820465C>T	c.2288G>A	p.(Gly763Asp)	16/20	rs371484780	1	0	0	1	0	0
17:59820468C>T	c.2285G>A	p.(Arg762His)	16/20	rs200960251	2	0	0	2	0	1
17:59820469G>A	c.2284C>T	p.(Arg762Cys)	16/20	rs587778136	0	1	0	1	0	0
17:59821817C>T	c.2233G>A	p.(Ala745Thr)	15/20	rs587780235	6	1	0	7	0	1
17:59821840T>C	c.2210A>G	p.(Glu737Gly)	15/20	rs755361298	1	0	0	1	0	0
17:59821871G>A	c.2179C>T	p.(Pro727Ser)	15/20	rs769797684	1	0	0	1	0	0
17:59821942T>A	c.2108A>T	p.(Lys703Ile)	15/20	rs756412722	2	1	0	3	0	0
17:59853769G>C	c.2090C>G	p.(Ser697Cys)	14/20	/	0	1	0	1	0	0
17:59853772G>A	c.2087C>T	p.(Pro696Leu)	14/20	rs147755155	5	0	0	5	0	1
17:59853887G>A	c.1972C>T	p.(Arg658Trp)	14/20	rs786203170	0	1	0	1	0	0
17:59853896G>A	c.1963C>T	p.(Pro655Ser)	14/20	rs753036322	1	0	0	1	0	0
17:59853907A>G	c.1952T>C	p.(Ile651Thr)	14/20	rs757305097	0	0	1	1	0	0
17:59853918C>A	c.1941G>T	p.(Trp647Cys)	14/20	rs786202760	0	1	0	1	0	0
17:59857651C>T	c.1906G>A	p.(Glu636Lys)	13/20	/	0	0	0	0	0	1
17:59857678G>A	c.1879C>T	p.(Leu627Phe)	13/20	/	0	0	0	0	0	1
17:59857707A>G	c.1850T>C	p.(Leu617Ser)	13/20	rs1064794095	0	0	1	1	0	0
17:59857749A>T	c.1808T>A	p.(Ile603Asn)	13/20	/	0	1	0	1	0	0
17:59857753C>A	c.1804G>T	p.(Asp602Tyr)	13/20	rs770750488	1	0	0	1	0	0
17:59857759A>G	c.1798T>C	p.(Phe600Leu)	13/20	rs745367580	1	0	0	1	0	0
17:59858214A>G	c.1781T>C	p.(Leu594Ser)	12/20	rs587781559	1	0	0	1	0	0
17:59861640T>A	c.1619A>T	p.(Gln540Leu)	11/20	rs4988349	1	0	0	1	0	0
17:59861755C>T	c.1504G>A	p.(Glu502Lys)	11/20	/	0	0	0	0	0	1

17:59876477A>G	c.1324T>C	p.(Cys442Arg)	09/20	/	0	1	0	1	0	0
17:59876545C>T	c.1256G>A	p.(Arg419Gln)	09/20	rs748105919	0	1	0	1	0	0
17:59876546G>A	c.1255C>T	p.(Arg419Trp)	09/20	rs150624408	16	5	7	28	3	15
17:59876594G>A	c.1207C>T	p.(Arg403Trp)	09/20	rs369631413	8	0	0	8	0	0
17:59876602T>C	c.1199A>G	p.(Asp400Gly)	09/20	/	0	0	0	0	0	1
17:59876603C>A	c.1198G>T	p.(Asp400Tyr)	09/20	rs764711572	1	1	0	2	0	3
17:59876617G>A	c.1184C>T	p.(Ala395Val)	09/20	/	0	0	0	0	0	1
17:59876648G>T	c.1153C>A	p.(Leu385Met)	09/20	/	0	0	0	0	0	1
17:59878615C>T	c.1139G>A	p.(Ser380Asn)	08/20	rs569696977	1	1	0	2	0	0
17:59878655A>G	c.1099T>C	p.(Cys367Arg)	08/20	/	0	0	0	0	0	1
17:59878699T>C	c.1055A>G	p.(Tyr352Cys)	08/20	rs762417690	1	0	0	1	0	0
17:59878700A>G	c.1054T>C	p.(Tyr352His)	08/20	rs730881632	1	1	0	2	0	0
17:59878714A>G	c.1040T>C	p.(Leu347Pro)	08/20	rs786201819	0	1	0	1	0	0
17:59878828G>A	c.926C>T	p.(Ser309Phe)	08/20	/	0	0	0	0	0	1
17:59885844A>G	c.902T>C	p.(Leu301Ser)	07/20	rs750376292	1	0	0	1	0	0
17:59885890G>A	c.856C>T	p.(Pro286Ser)	07/20	rs770289817	1	0	0	1	0	0
17:59885893G>A	c.853C>T	p.(His285Tyr)	07/20	/	0	0	0	0	0	1
17:59885898C>T	c.848G>A	p.(Cys283Tyr)	07/20	rs771096783	1	0	0	1	0	0
17:59885926T>C	c.820A>G	p.(Thr274Ala)	07/20	rs62620988	8	0	0	8	0	0
17:59885994C>T	c.752G>A	p.(Arg251His)	07/20	rs780834054	3	0	0	3	0	0
17:59885995G>A	c.751C>T	p.(Arg251Cys)	07/20	rs752309409	1	1	0	2	0	1
17:59886006T>C	c.740A>G	p.(Tyr247Cys)	07/20	rs756499865	1	0	0	1	0	0
17:59886096C>A	c.650G>T	p.(Cys217Phe)	07/20	rs587782156	0	0	0	0	0	1
17:59924539C>A	c.550G>T	p.(Asp184Tyr)	06/20	rs201047375	17	2	2	21	0	6
17:59924571C>T	c.518G>A	p.(Arg173His)	06/20	rs761432927	1	0	0	1	0	0
17:59924572G>A	c.517C>T	p.(Arg173Cys)	06/20	rs4988345	212	84	14	310	9	49
17:59926512C>T	c.485G>A	p.(Arg162Gln)	05/20	rs61757643	1	1	0	2	0	0
17:59926524T>G	c.473A>C	p.(Lys158Thr)	05/20	rs769364081	1	0	0	1	0	0
17:59926549C>T	c.448G>A	p.(Glu150Lys)	05/20	rs762701532	2	0	0	2	0	0
17:59937199C>T	c.163G>A	p.(Ala55Thr)	03/20	rs757909937	1	0	0	1	0	0
17:59937223G>C	c.139C>G	p.(Pro47Ala)	03/20	rs28903098	23	5	3	31	3	10
17:59937241G>A	c.121C>T	p.(His41Tyr)	03/20	rs770930270	1	0	0	1	0	0
17:59938819T>C	c.82A>G	p.(Met28Val)	02/20	/	0	0	0	0	0	1
**Cumulative carrier frequency (%)**					341/27,173 **(1.25%)**	115/7,325 **(1.56%)**	29/2,189 **(1.32%)**	485/36,687 **(1.32%)**	20/706 **(2.83%)**	102/6,341 **(1.61%)**

Table S4: Potentially damaging missense variants identified in the *BRIP1* gene (transcript NM_032043.2). Missense variants were defined as potentially damaging when predicted deleterious by the *in silico* tools SIFT and MutationTaster (Alamut version 2.10 as November 9th 2017). A total of 341 distinct potentially damaging missense variants were listed in the ExAC database (Exome Aggregation Consortium, non-Finnish Europeans; excluding The Cancer Genome Atlas data; as of June 2016); 115 were listed in the FLOSSIES database (American-European ancestry) and 29 were identified in geographically-matched female controls (GMCs); 122 distinct potentially damaging missense variants were found in 7,047 familial BC/OC patients.
